# Supplementary figures and images for: Bioinformatic Analyses of Peripheral Blood Transcriptome Identify Altered Neutrophil-Related Pathway and Different Transcriptomic Profiles for Acute Pancreatitis in Patients with and without Chylomicronemia Syndrome
Source: Biomolecules. 2023 Feb 2;13(2):284. doi: 10.3390/biom13020284 (PMC9953624; doi:10.3390/biom13020284)

# Degree

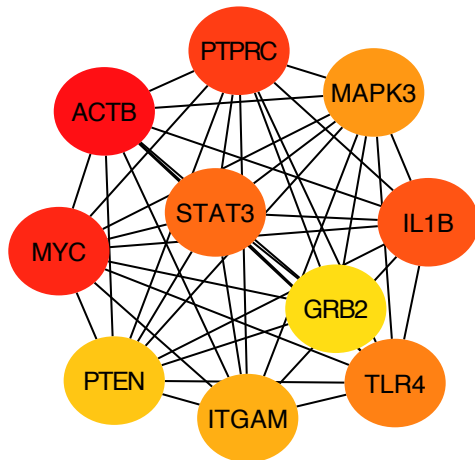

# DMNC

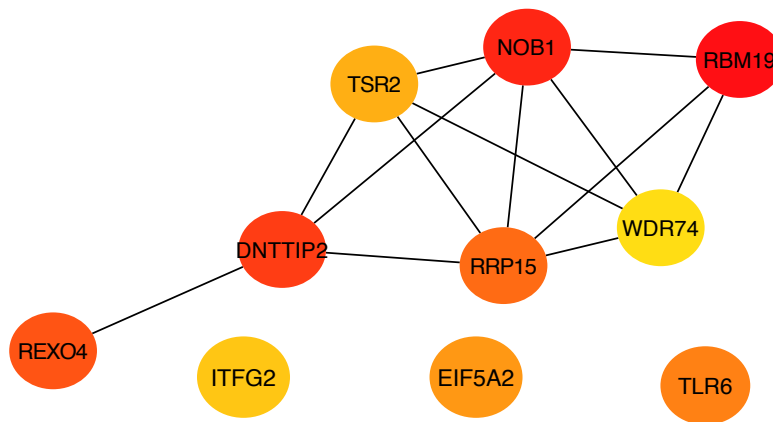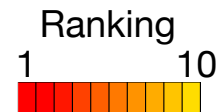

# EPC

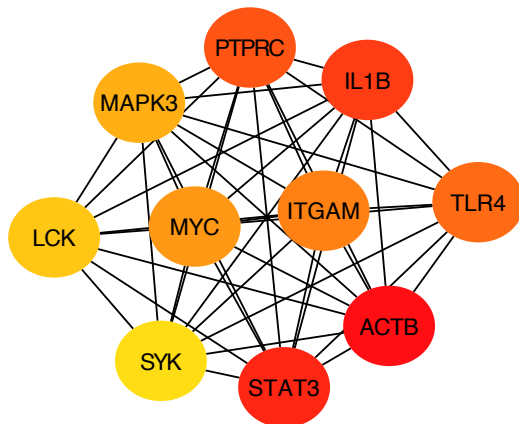

# MCC

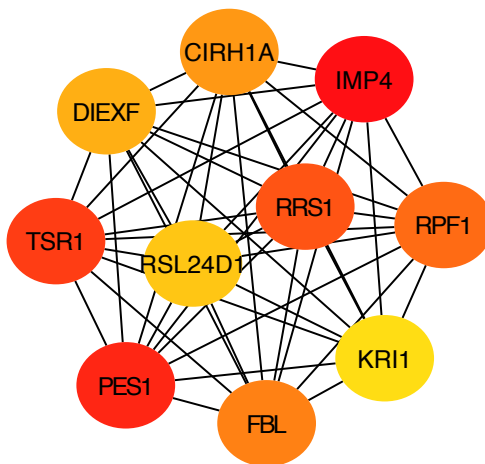

# MNC

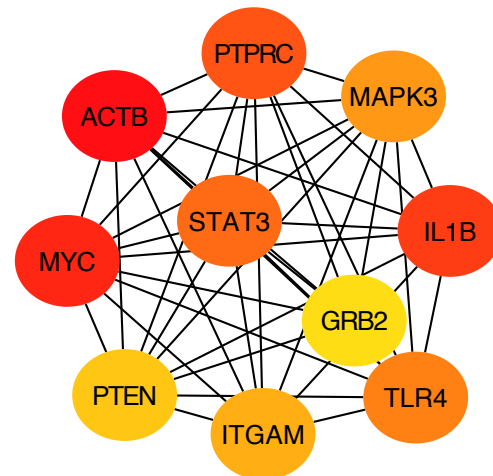

Supplement: Supplementary file 1 [file biomolecules-13-00284-s001.zip › FigureS2.pdf]

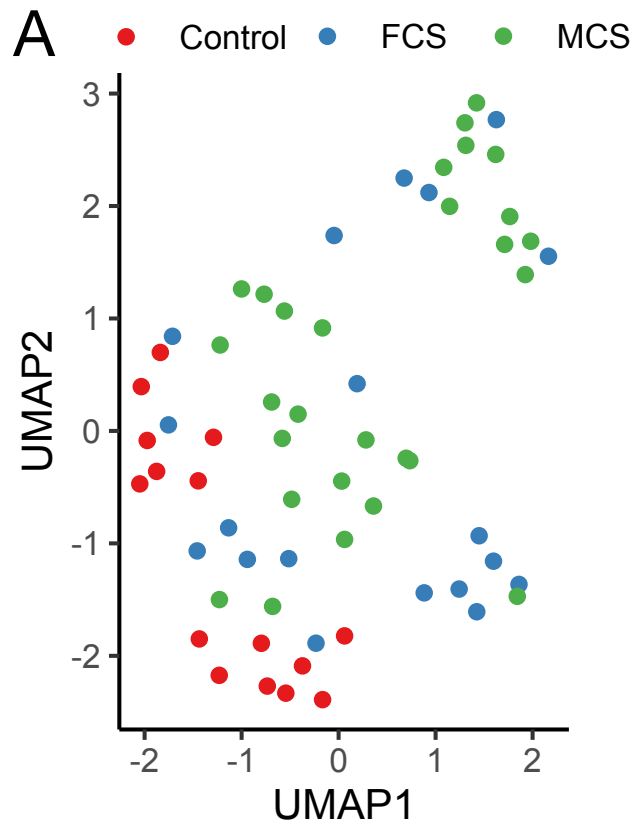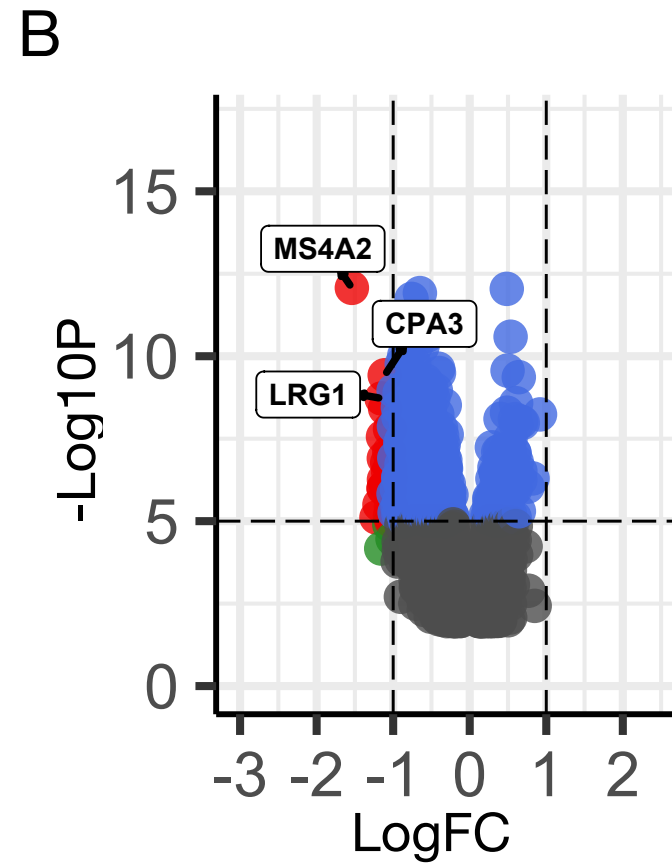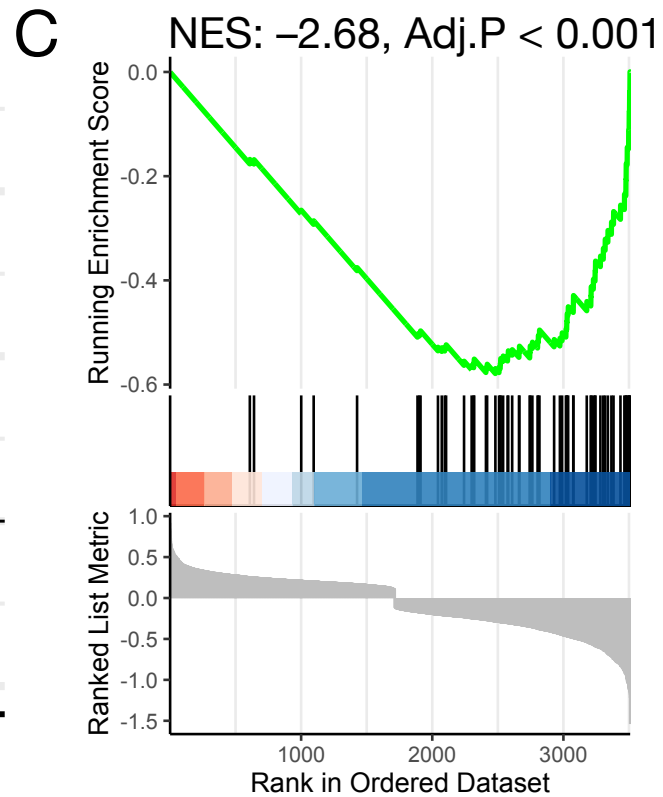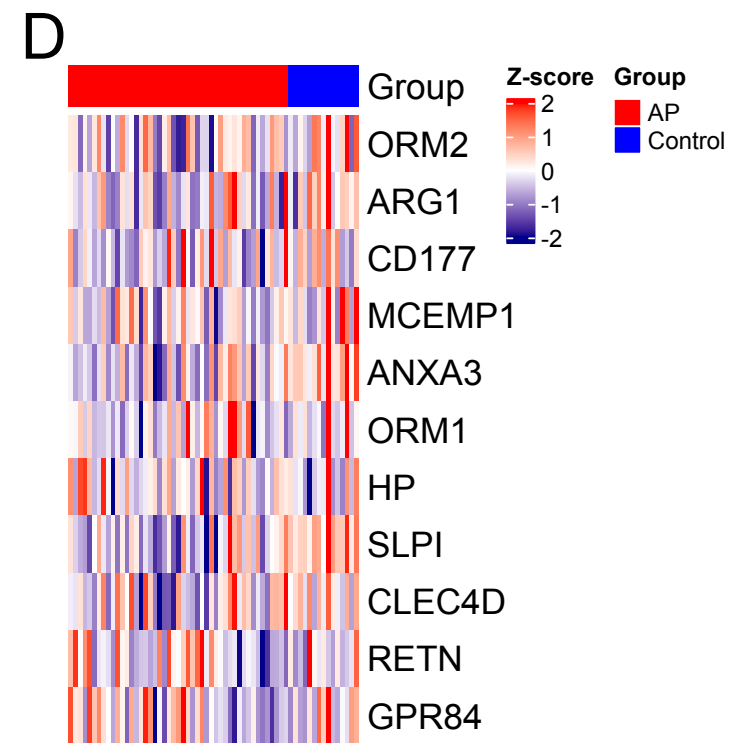

Supplement: Supplementary file 1 [file biomolecules-13-00284-s001.zip › FigureS1.pdf]
